# *CHD7* variants associated with hearing loss and enlargement of the vestibular aqueduct

**DOI:** 10.1007/s00439-023-02581-x

**Published:** 2023-09-05

**Authors:** Isabelle Roux, Cristina Fenollar-Ferrer, Hyun Jae Lee, Parna Chattaraj, Ivan A. Lopez, Kyungreem Han, Keiji Honda, Carmen C. Brewer, John A. Butman, Robert J. Morell, Donna M. Martin, Andrew J. Griffith

**Affiliations:** 1 Otolaryngology Branch, National Institute on Deafness and Other Communication Disorders (NIDCD), National Institutes of Health (NIH), Bethesda, MD 20892 USA; 2 Laboratory of Molecular Genetics, NIDCD, NIH, Bethesda, MD 20892 USA; 3 The NIDCD National Temporal Laboratory at UCLA, Department of Head and Neck Surgery, David Geffen School of Medicine at UCLA, Los Angeles, CA 90095 USA; 4 Laboratory of Membrane Biophysics, NHLBI, National Institutes of Health, Bethesda, MD 20892 USA; 5 Department of Otorhinolaryngology, Tokyo Medical and Dental University, Bunkyo-ku, Tokyo, Japan; 6 Radiology and Imaging Sciences, Clinical Center, NIH, Bethesda, MD 20892 USA; 7 Genomics and Computational Biology Core, NIDCD, NIH, Bethesda, MD 20892 USA; 8 Department of Human Genetics, University of Michigan Medical School, Ann Arbor, MI USA; 9 Department of Pediatrics, University of Michigan Medical School, Ann Arbor, MI USA; 10 Department of Otolaryngology, College of Medicine, University of Tennessee Health Science Center, Memphis, TN 38163 USA

## Abstract

Enlargement of the endolymphatic sac, duct, and vestibular aqueduct (EVA) is the most common inner ear malformation identified in patients with sensorineural hearing loss. EVA is associated with pathogenic variants in *SLC26A4.* However, in European–Caucasian populations, about 50% of patients with EVA carry no pathogenic alleles of *SLC26A4*. We tested for the presence of variants in *CHD7*, a gene known to be associated with CHARGE syndrome, Kallmann syndrome, and hypogonadotropic hypogonadism, in a cohort of 34 families with EVA subjects without pathogenic alleles of *SLC26A4*. In two families, NM_017780.4: c.3553A > G [p.(Met1185Val)] and c.5390G > C [p.(Gly1797Ala)] were detected as monoallelic *CHD7* variants in patients with EVA. At least one subject from each family had additional signs or potential signs of CHARGE syndrome but did not meet diagnostic criteria for CHARGE. In silico modeling of these two missense substitutions predicted detrimental effects upon CHD7 protein structure. Consistent with a role of CHD7 in this tissue, *Chd7* transcript and protein were detected in all epithelial cells of the endolymphatic duct and sac of the developing mouse inner ear. These results suggest that some *CHD7* variants can cause nonsyndromic hearing loss and EVA. *CHD7* should be included in DNA sequence analyses to detect pathogenic variants in EVA patients. *Chd7* expression and mutant phenotype data in mice suggest that CHD7 contributes to the formation or function of the endolymphatic sac and duct.

**Supplementary Information:**

The online version contains supplementary material available at 10.1007/s00439-023-02581-x.

## Introduction

Unilateral or bilateral enlargement of the vestibular aqueduct (EVA) is detected in 5–15% of children with sensorineural hearing loss (SNHL) whose temporal bones are evaluated with computed tomography (CT) or magnetic resonance imaging (MRI) (reviewed in (Griffith and Wangemann 2011)). Audiometry of ears with EVA demonstrates SNHL with an occasional conductive component at low frequencies in the presence of normal middle ear structure and function. This conductive component is caused by a biomechanical effect of EVA upon sound transmission within the inner ear (Merchant et al. 2007).

Patients with EVA often present with Pendred syndrome, defined as bilateral EVA with an iodine organification defect that can lead to thyroid goiter (MIM:274600) (Madeo et al. 2009). Pendred syndrome is caused by biallelic pathogenic variants in the *SLC26A4* gene (MIM:605646) (Everett et al. 1997). Nonsyndromic recessive EVA associated with *SLC26A4* pathogenic variants is referred to as DFNB4 (MIM:600791). Pathogenic variants of *SLC26A4* are found in 40–50% of European–Caucasian patients with EVA. Biallelic pathogenic variants in *FOXI1* (MIM:601093) are a rare cause of EVA associated with distal renal tubular acidosis (Enerback et al. 2018).

The causes of EVA in patients without pathogenic alleles of *SLC26A4*, termed M0 patients, remain largely unknown. We have previously reported a very low probability of EVA in non-twin siblings of M0 EVA probands, suggesting that many, if not most, cases have a complex genetic or non-genetic etiology (Choi et al. 2009a).

EVA associated with SNHL has been found sporadically, most often unilaterally, in a few patients who fulfill the diagnostic criteria of branchio-oto-renal (BOR) syndrome, branchio-otic (BO) syndrome, Waardenburg syndrome, or CHARGE syndrome. These dysmorphogenetic syndromes segregate as dominant traits with incomplete penetrance and variable expressivity in most families. Syndromic forms of SNHL sometimes mimic nonsyndromic SNHL, presenting with subtle syndromic findings which do not meet the criteria for formal diagnosis (Bademci et al. 2016). However, there are currently no reports of nonsyndromic EVA as a potential allelic disorder with BOR, BO, Waardenburg, or CHARGE syndromes.

The major acronymous features of CHARGE syndrome (MIM:214800) are: **c**oloboma of the eye, **h**eart defects, **a**tresia of the choanae, **r**etarded growth and development, **g**enitourinary abnormalities and external and internal **e**ar anomalies (Blake et al. 1998; Hale et al. 2016; Verloes 2005). *CHD7* (MIM:608892) pathogenic variants are a common cause of CHARGE syndrome (Pagon et al. 1981; Vissers et al. 2004). Agenesis or hypoplasia of the semicircular canals is the most common malformation of the inner ear, identified in 95% of 35 patients with a *CHD7* pathogenic variant (Bergman et al. 2012b). In contrast, unilateral or bilateral EVA has only been detected in a few patients with CHARGE syndrome (Abadie et al. 2000; Bedeschi et al. 2020; Hoch et al. 2017; Vesseur et al. 2016). Other signs of CHARGE include external ear anomalies, hypogonadotropic hypogonadism characterized by absent, partial or delayed puberty, anosmia or hyposmia, renal or collecting system malformations, cleft lip and/or cleft palate, cranial nerve dysfunction, and atopic disorders (Kong and Martin 2018). *CHD7* variants can also cause Kallmann syndrome and isolated hypogonadotropic hypogonadism (Bergman et al. 2012a; Jongmans et al. 2009; Kim et al. 2008; Marcos et al. 2014).

*CHD7* codes for chromodomain helicase DNA-binding protein 7 (CHD7), a transcriptional regulator that acts as an ATP-dependent chromatin remodeler and an epigenetic regulator. It contains two chromodomains, a centrally located SNF2-like ATPase motor formed by two lobes, a SANT–SLIDE domain that binds DNA, and two BRK domains. Despite the importance of this protein, three-dimensional structural information is limited to the NMR-derived structure of the BRK domains located at its C-terminal end (Allen et al. 2007), which made it impossible to study the structural consequences of most pathogenic variants.

During the development of the mouse inner ear, *Chd7* expression can be detected from embryonic day 9.5 (E9.5). It is present in both the dorsal and ventral otocyst at E10.5, before becoming progressively restricted to the sensory epithelia of the vestibule and cochlea, and the vestibulo-cochlear ganglion (Bosman et al. 2005; Hurd et al. 2010). This expression persists, with *Chd7* being highly expressed in mature auditory and vestibular hair cells and in spiral ganglion neurons (Hurd et al. 2011). However, the longitudinal temporal expression of *Chd7* in the endolymphatic duct and sac, which begin developing from the dorsomedial region of the otocyst at E10.75 (Hultcrantz et al. 1987; Morsli et al. 1998), has not been described.

Here we sought to identify potentially damaging variants in *CHD7* segregating among a cohort of 34 M0 families with EVA. We generated a structural model of CHD7 in its active and inactive forms and determined that two of the identified variants in *CHD7* are predicted to induce abnormal local folding of the protein and are likely to be pathogenic. We also show that *Chd7* is expressed in the endolymphatic sac and duct of the developing mouse inner ear. Our results indicate that some *CHD7* variants are associated with SNHL and EVA without major semicircular canal defects as part of an atypical CHARGE phenotype.

## Materials and methods

### Human subjects

The study cohort included 34 families with 51 subjects (28 females, 23 males) with EVA. Thirty-seven subjects had bilateral EVA and 14 had unilateral EVA. EVA was transmitted from parent to child in two families (372 and 388) (Muskett et al. 2016). Some subjects have been described (Chattaraj et al. 2017; Choi et al. 2009a, b; Muskett et al. 2016; Pryor et al. 2005). Race and ethnicity were classified according to our institutional review board reporting guidelines. Thirty-two families were European–Caucasian and non-Hispanic/Latino, one family was African American/Black, and one was multiracial. None of the subjects with EVA met diagnostic criteria for BOR, BO, Waardenburg or CHARGE syndromes, nor were these diagnoses reported for any family member. Subjects had no pathogenic variants detected by Sanger sequence analysis of the single protein-coding exon of *GJB2* (MIM:121011). A vestibular aqueduct was initially defined as enlarged (EVA) if its diameter was greater than 1.5 mm at the midpoint between the posterior cranial fossa and the vestibule of the inner ear. This criterion was subsequently reduced to > 1.0 mm, which is now the standard (Muskett et al. 2016). An EVA subject and their family were included only if the subject was M0, i.e., did not carry biallelic pathogenic variants in the coding regions or splice sites of *SLC26A4* (M2) and did not carry one such variant (M1) in *trans* with an allele with the CEVA (“Caucasian EVA”) haplotype (Chattaraj et al. 2017; Choi et al. 2009a)*.* In family 388, all individuals with EVA carry the likely benign *SLC26A4* variants NM_000441.2:c.-66C > G and c.970A > T; p.(Asn324Tyr) in *cis*. However, no other variants in *SLC26A4* nor the CEVA haplotype were identified in *trans* in these individuals. In family 258, subject 1693 is homozygous for CEVA. In family 276, individual 1810 does not carry CEVA.

### Clinical evaluation

Subjects from 33 of 34 study families (except family 400) were evaluated at the NIH Clinical Center as previously reported (Choi et al. 2009a; Muskett et al. 2016). Eight subjects were adults, 41 were between 1 year and 17 years, with a mean (standard deviation) age of 7.0 (± 3.7) years at the time of the evaluation for this study at the NIH. Subjects 2136 and 2137 from family 400 were 16 and 13 years, respectively, when they enrolled in this study. Evaluations included pure-tone and immittance audiometry, and CT or MRI scans of the temporal bones. CT and MRI scans were analyzed by the same neuroradiologist (J.A.B.) and otolaryngologist—head and neck surgeon (A.J.G.). Normal hearing was defined as air-conduction thresholds less than or equal to 15 dB HL for subjects less than 18 years, and less than or equal to 25 dB HL at six octave test frequencies (250 Hz to 8 kHz) for adult subjects. For study subjects with SNHL but no temporal bone imaging results, we considered their SNHL to be inconsistent with EVA if it had an onset during adulthood or was otherwise phenotypically distinguishable from the range of auditory phenotypes associated with EVA (Griffith and Wangemann 2011).

### Nucleotide sequence analysis

Genomic DNA (gDNA) was extracted from peripheral blood samples using the Gentra Puregene Blood Kit (Qiagen, Germantown, MD, USA) and quantified using a Nanodrop spectrophotometer and a Qubit 4 Fluorometer (Thermo Fisher Scientific, Waltham, MA, USA). Exome sequencing was performed with gDNA from 47 of 51 subjects with EVA. Genomic DNA from subject 1598 was not available and only one gDNA sample was included for each of three pairs of monozygotic twins whose zygosity status had been confirmed by molecular testing. Exome libraries were prepared using a Nextera Rapid Capture Exome kit (Illumina, San Diego, CA, USA) and sequenced on an Illumina NextSeq500 instrument. Libraries were sequenced in groups of 12 uniquely barcoded samples with an average coverage depth of greater than 100X.

Sequence reads were mapped to the GRCh38 human reference genome using the bcbio-nextgen germline variant calling pipeline (https://bcbio-nextgen.readthedocs.io/en/latest/contents/pipelines.html#germline-variant-calling). Reads were mapped using the Burrows–Wheeler Aligner (BWA–MEM), then remapped after deduplication and GATK (Genome Analysis Toolkit) recalibration. Variants were detected using GATK-haplotypecaller, platypus, varscan, freebayes and samtools variant callers, and the final ensemble variant call file (vcf) required concordance between at least two callers.

The presence of potential damaging variants in *CHD7* was investigated using Ingenuity Variant Analysis platform (Qiagen). Variants predicted to affect canonical splice sites, insertion–deletions, nonsense and missense variants with combined annotation dependent depletion (CADD) score (Rentzsch et al. 2019) of at least 20 were further considered (Table 1). Potential pathogenicity was further analyzed in silico using REVEL (Ioannidis et al. 2016), SIFT (Kumar et al. 2009), PolyPhen (Adzhubei et al. 2010), FATHMM-XF dbNSFP 4.2 (Rogers et al. 2018; Shihab et al. 2015), and Mutation Taster (Schwarz et al. 2010). Evolutionary conservation of residues in vertebrates was evaluated by phyloP (Pollard et al. 2010). The presence and familial segregation of variants was confirmed by Sanger sequencing using an ABI3730XL genetic analyzer (Applied Biosystems, Foster City, CA, USA). Sequences of all primers used in this study are available in Table S1. The presence of copy number variants and large chromosomal rearrangements was not investigated.

**Table 1:** Seven variants were identified in *CHD7* in seven families with bilateral sensorineural hearing loss and bilateral enlargement of the vestibular aqueduct (EVA)

Nucleotide variant* Amino acid variation	Genomic position**	Family	Subject (age at examination in years)	Family ancestry	REVEL score	CADD score GRCh38-v1.6	SIFT	PolyPhen function prediction	FATHMM—XF	Mutation Taster	Conservation vertebrate phyloP	gnomAD MAF in the corresponding population^†^ (number of alleles sequenced)/Maximum MAF in any population if higher (number of alleles sequenced)	ACMG/AMP variant classification according to (Oza et al. 2018) (ACMG criteria)
c.307 T > A p.(Ser103Thr)	60,741,739	258	1693 (25)	European	0.063 Benign	22.9	Damaging	Benign	Neutral	Disease causing	4.582	0.01908 (68,020)	Benign (BA1, BP4)
c.2230G > A p.(Gly744Ser)	60,795,119	281	1817 (5)	Caucasian	0.2919 Benign	24.4	Tolerated	Probably Damaging	Damaging	Disease causing	9.019	0.0000294 (68,034)/ 0.01529 in Africans/African Americans (41,408)††	Benign (BA1)
c.3553A > G p.(Met1185Val)	60,830,352	276	1810 (6)	Jewish Ashkenazi	0.883 Patho-genic	24.9	Damaging	Possibly Damaging	Damaging	Disease causing	6.919	Not found (3468)	Variant of Uncertain Significance (PM2_sup, PP3)
c.5390G > C p.(Gly1797Ala)	60,849,140	388	2106 (41) 2107 (8) 2108 (8)	European	0.8989 Patho-genic	28.0	Damaging	Probably Damaging	Damaging	Disease causing	11.295	0.0000147 (68,032)/ 0.00002415 in Africans/African Americans (41,416)	Likely Pathogenic (PS4_supporting, PM2_sup, PM5, PP3)
c.8950C > T p.(Leu2984Phe)	60,865,889	388	2107 (8) 2108 (8)	European	0.06499 Benign	16.55	Tolerated	Benign	Neutral	Poly-morphism	4.015	0.004777 (68,036) /0.013 in European (Finnish) (10,612)	Benign (BA1, BP4)
c.7855 T > C p.(Ser2619Pro)	60,862,220	234	1634 (7)	Caucasian	0.155 Benign	22.5	Tolerated	Benign	Neutral	Disease causing	3.453	0.00004409 (68,036)	Variant of Uncertain Significance (BP4)
c.2053_2058dupGCAAAA p.(Ala685_ Lys686dup)	60,781,383	284 347	1832 (6) 2044 (17)	European	N/A	20.5	N/A	N/A	N/A	N/A	N/A	0.01192 (68,038)/ 0.05811 in Amish (912)	Benign (BA1, BS4, BP3)

None of these variants were predicted to alter splicing according to SpliceAI, except for c.7855 T > C which had a score of 0.0200 for SpliceAI (acceptor gain)

N/A predictions not available

Databases last checked on 11/25/2022

*Based on NM_017780.4

**Based on hg38 assembly of chromosome 8

^†^Maximum minor allele frequency (MAF) from corresponding populations from gnomAD v3.1.2 with results from 76,156 genomes from unrelated individuals sequenced as part of various disease-specific and population genetic studies

^††^Variant found at an MAF of 0.060 (11/173 allele) and 0.061 (12/184 alleles) in the Gambian Genome Variation Project in Gambian in the Western Divisian, The Gambia–Fula and Wolof, respectively (information available through Ensembl)

The presence of NM_017780.4:c.3553A > G among Ashkenazi Jewish was investigated in IBD Exomes Portal, Cambridge, MA, USA (https://dmz-ibd.broadinstitute.org/http://ibd.broadinstitute.org) (accessed 09/08/2022) and was also tested by Sanger sequencing a panel of 218 gDNA samples from The National Laboratory for the Genetics of Israeli Populations. Variants were classified according to the American College of Medical Genetics and Genomics/Association for Molecular Pathology (ACMG/AMP) Guidelines for the interpretation of sequence variants in hearing loss genes (Oza et al. 2018).

### Computational modeling of CHD7 structure

Hidden Markov model profiles (HMMs) were used as a descriptor for human CHD7 amino acid sequence to identify a suitable structural template for modeling. The HMM profile obtained after a three-iteration sequence scanning performed using UniRef30 sequence database was subsequently scanned against the HMMs of the sequences corresponding to each of the Protein Data Bank (PDB) X-ray structures (pdb70 database) as per standard procedure in HHpred server (Hildebrand et al. 2009; Zimmermann et al. 2018). As *Saccharomyces cerevisiae* CHD1 in active (PDB id: 5o9g) and inactive (PDB id: 3mwy) states (Farnung et al. 2017; Hauk et al. 2010) had the highest coverage (614 residues), the highest sequence identity (35%) and best correspondence between secondary structural elements, they were selected as templates for structural modelling. Contrary to the active structure, which contains a histone complex and double-stranded DNA fragment in addition to two chromodomains, the ATPase motor (lobes 1 and 2) and SANT–SLIDE domains, the inactive structure only contains the two chromodomains and the ATPase motor (lobes 1 and 2).

The initial sequence alignment between CHD1 and CHD7 obtained with HHpred was refined in an iterative process that used conservation scores obtained with Consurf server (Ashkenazy et al. 2016) as a guide. This procedure positions the most conserved residues packing towards the core of the protein and removes gaps, as needed, within secondary structural elements. The final alignment was used during the modeling production run of active and inactive states, where 2000 modeling iterations per run were performed with MODELLER (Webb and Sali 2016). For each state, the model with highest MODELLER probability distribution function score (molPDF) and best stereochemistry as per Procheck analysis (Laskowski et al. 1993) was selected as the final wild-type active and inactive models. A similar procedure was used to obtain structural models of CHD7 in active and inactive states with the p.(Met1185Val) and p.(Gly1797Ala) variants. For modelling of the inactive state, the SANT–SLIDE domain was added to the inactive structure by structurally superimposing lobe 1 in the ATPase motor from the active structure onto lobe 1 of the inactive structure (Fig. S3A).

### Animal procedures

C57BL/6 J mice (stock number #000664) and *Chd7*^*Gt(S20−7E1)Sor/+*^ mice (B6;129S-*Chd7*^*Gt(S20−7E1)Sor*^/DmmJ #030659) were obtained from the Jackson Laboratory (Bar Harbor, ME, USA). In *Chd7*^*Gt(S20−7E1)Sor/+*^ mice, the ROSAFARY gene trap vector has been inserted between exon 1 and 2, leading to a functional null allele of *Chd7* with disruption of splicing and generation of fusion transcripts of exon1/β-galactosidase and hygromycin cassette/exon 2 (Hurd et al. 2007). The first day after overnight mating was counted as E0.5. The sex of the embryos was determined by PCR (Tunster 2017).

### Histochemistry and immunohistochemistry

The posterior region of hemi-skull containing the inner ear was harvested from mice at E14.5 to postnatal day 30 (P30), quickly immersed in ice cold 4% paraformaldehyde (Electron Microscopy Sciences, Hatfield, PA) freshly diluted in phosphate-buffered saline (PBS), and incubated for 15 min (for X-gal staining) or 1 h (for immunohistochemistry) at 4 $^\circ{\rm C}$, before being washed three times for 30 min in PBS (Honda et al. 2021).

The vestibular aqueduct, endolymphatic sac and adjacent tissue were quickly separated from the rest of the inner ear, incubated with filtered, prewarmed X-gal solution (1 mg/ml X-gal/5% *N*,*N*-dimethylformamide, 5 mM potassium ferricyanide [K_3_Fe(CN)_6_], 5 mM potassium ferrocyanide trihydrate [K_4_Fe(CN)_6_–3H_2_O], and 2 mM MgCl_2_ diluted in PBS (pH 7.3) with 0.02% NP40 and 0.1% sodium deoxycholate) with gentle agitation overnight at 37 $^\circ{\rm C}$, protected from light. After three washes in PBS, the preparations were postfixed with 4% paraformaldehyde diluted in PBS for 1 h 30 min at room temperature (RT). After three washes in PBS, the endolymphatic sac and duct were further microdissected to eliminate as much adjacent tissue as possible before mounting on slides using FluorSave mounting medium (Calbiochem, San Diego, CA, USA). Samples were imaged using a Zeiss Imager Z1 microscope (Carl Zeiss Microimaging, Thornhood, NY) with differential interference contrast optics, a Fluar 10x objective and a Plan-Apochromat 63x/1.40 N.A. oil objective, coupled to an Axiocam 712 color camera and Zen 3.1 blue edition software (Zeiss).

For immunolabeling, after fixation, the endolymphatic sac and duct were first microdissected from the adjacent tissue (Honda et al. 2021). The preparations were then permeabilized for 30 min in PBS with 0.5% Triton X-100, blocked for 1 h at RT in PBS containing 1% BSA and 10% donkey serum, then incubated with the primary antibodies diluted in the same solution for 24 h at 4 $^\circ{\rm C}$. Rabbit monoclonal anti-CHD7 (D3F5, #6505S, LOT: 1, Cell signaling Technology, Danvers, MA, USA, raised against a recombinant protein corresponding to the N-terminal region of human CHD7 protein), Alexa Fluor 594-conjugated rabbit anti-CHD7 (#NBP1-77393AF594, LOT: A-030520-AF594, Novus, Littleton, CO, USA, raised against the N-terminal region of the human CHD7 protein (within residues 25–200) [Swiss-Prot Q9P2D1]), and goat anti-FOXI1 (RRID:AB_732416, ab20454, Abcam, Cambridge, MA, USA) antibodies were used as primary antibodies at 1:50, 1:200 and 1:200 dilutions, respectively. After three 30-min washes in PBS, the samples were incubated for 1 h at RT with the secondary antibodies diluted 1:1000 in blocking solution. Alexa Fluor 488-conjugated donkey anti-goat and Alexa Fluor 568-conjugated donkey anti-rabbit antibodies (Molecular Probes, Eugene, OR, USA) were used as needed as secondary antibodies. Samples were washed three times for 30 min in PBS with agitation at RT. Counterstaining with Hoechst 33342 (1:200 in PBS, H3570, Lot: 1,985,413, Invitrogen, Waltham, MA, USA) was performed for 10 min at RT. After three 30-min washes, samples were mounted in a drop of FluorSave mounting medium on slides and imaged using a LSM 880 confocal microscope (Zeiss) with a Plan-Apochromat 63x/1.4 N.A. oil objective. Stitching for the endolymphatic sac overview, reconstructions and analysis were carried out using Zen software (Zeiss).

## Results

### *CHD7* variants identified in families with SNHL and EVA

**Fig. 1:**
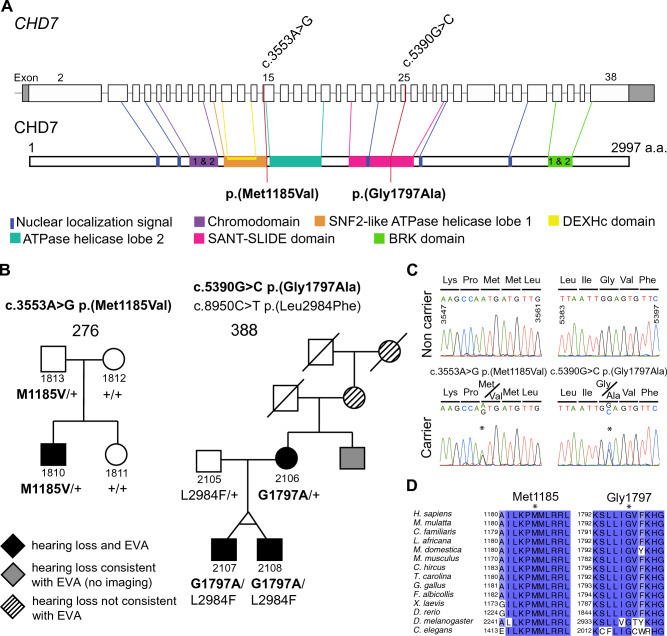
*CHD7* variants and inner ear phenotypes. **A** Schematic representation of the coding exons of *CHD7* and reported functional domains of the CHD7 protein. The variants NM_017780.4: c.3553A > G [p.(Met1185Val)] and c.5390G > C [p.(Gly1797Ala)] were identified in our EVA cohort in families 276 and 388, and are likely causal. The other variants presented in Table 1 not thought to be pathogenic are not shown. **B** Segregation of EVA, SNHL, and *CHD7* variants in families 276 and 388. Variants in bold are thought to be causal. One subject in family 388, who did not enroll in the study, was reported to have had bilateral hearing loss since 10 years of age which progressed to severe levels requiring bilateral hearing aids by the age of 39 years. This functional phenotype was not evaluated by radiologic imaging but was considered to be consistent with EVA (grey symbol). Other subjects had reported hearing loss whose onset and progression in adulthood was more consistent with aging as the primary etiology (presbycusis) rather than EVA (striped symbol). The genotype is indicated for all the gDNA available. “ + ” denotes a wild-type allele. **C** Chromatograms showing the presence of the c.3553A > G and c.5390G > C heterozygous variants of *CHD7* detected in families 276 and 388. **D** Conservation of human CHD7 amino acid residues Met1185 and Gly1797 among vertebrate and invertebrate (*Drosophila melanogaster*, *Caenorhabditis elegans*) species. Blue highlighting reflects conservation among 11 (light blue) or 13–14 (dark blue) of the 14 orthologs shown. Sequences obtained through Uniprot website were analyzed in Jalview. Alignment was performed using ClustalW. Protein sequences identifiers for CHD7 orthologues are Q9P2D1 for *Homo sapiens*, F6PP91 for *Macaca mulatta*, F1PWD8 for *Canis familiaris*, G3UE09 for *Loxodonta africana*, F7G444 for *Monodelphis domestica*, A2AJK6 for *Mus musculus*, A0A452E916 for *Capra hircus*, A0A674K692 for *Terrapene carolina triunguis*, Q06A37 for *Gallus gallus*, U3JST3 for *Ficedula albicollis*, A0A1L8FT46 for *Xenopus laevis*, F1QGL1 for *Danio rerio*, M9NEL3 for *Drosophila melanogaster*, O61845 for *Caenorhabditis elegans*

Seven EVA subjects from five families (234, 258, 276, 281 and 388) had missense variants in *CHD7* with a CADD score above 20 (Table 1, Figs. 1 and S1). The variants were all located in different exons of *CHD7* and are present in different functional domains of the corresponding protein (Fig. 1A).

All seven subjects presented with bilateral SNHL and bilateral EVA without semicircular canal hypoplasia or malformation. Each of the families segregated a different missense variant and one family (388) segregated two private missense variants. A duplication variant, c.2053_2058dupGCAAAA, p.(Ala685_Lys686dup), was identified in two subjects (1832 and 2044) from two other families (284 and 347, respectively). Affected subjects inherited the *CHD7* variant from an unaffected parent in six of seven families (Figs. 1 and S1). In family 388, affected monozygotic twin brothers (2107 and 2108) and their affected mother (2106) all carried the c.5390G > C, p.(Gly1797Ala) variant. The parents of 2106 did not participate in the study so it is unknown if this variant was inherited or arose de novo.

Only the variants in families 276 and 388 were both rare (MAF in any population < 0.01), affected residues conserved through evolution and were consistently predicted to have deleterious effects by in silico analysis, with a REVEL score > 0.7 (Table 1, Figs. 1D, S2).

#### Family 276

This family of Ashkenazi Jewish descent was previously reported (Chattaraj et al. 2017; Choi et al. 2009b). The EVA proband, subject 1810, of family 276 carried the variant c.3553A > G, p.(Met1185Val), which has a REVEL score of 0.883 and a CADD score of 24.9 (Table 1, Fig. 1B, C). In silico predictions of its pathogenic potential were damaging (SIFT), possibly damaging (PolyPhen), damaging (FATHMM-XF), and disease-causing (Mutation Taster). Methionine is conserved at this position among orthologs of CHD7 (Fig. 1D). p.(Met1185Val) was not identified in the samples included in gnomAD v3.1.2, nor in 5685 samples from individuals of Ashkenazi Jewish descent (IBD Exomes Portal), nor in a panel of 218 Ashkenazi Jewish control samples from The National Laboratory for the Genetics of Israeli Populations.

**Fig. 2:**
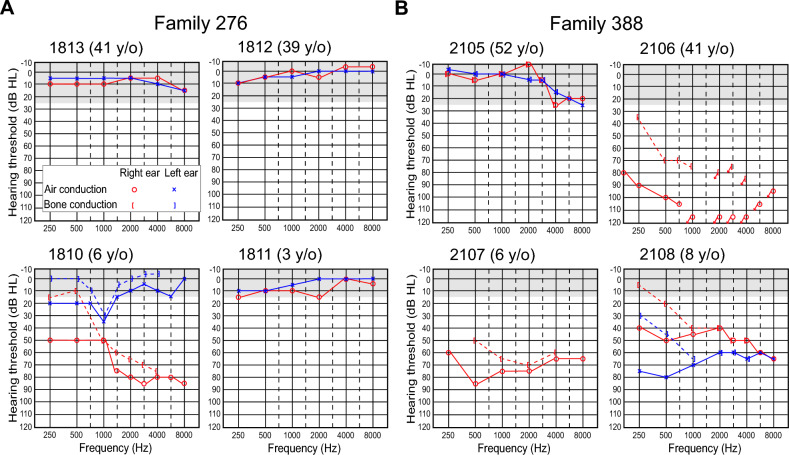
Audiograms for members of families 276 and 388.** A** Family 276: the audiogram of subject 1810 indicated a bilateral, asymmetric hearing loss. In the left ear, there was a slight-to-mild conductive hearing loss limited to the low-to-mid frequencies. In the right ear, he had a moderate low frequency conductive loss and a moderate-to-severe, mid-to-high frequency mixed hearing loss that was primarily sensorineural. Audiograms from subjects 1813, 1812 and 1811 indicated normal hearing sensitivity bilaterally. **B** For family 388: hearing sensitivity for subject 2105 was within normal limits. Subject 2106 had severe-to-profound mixed hearing loss in the low frequencies and no measurable hearing at 1000 Hz and above in her right ear. Her left ear was not tested as she had a cochlear implant. For subject 2107, his most recent audiogram before bilateral cochlear implantation is presented. There was a moderate-to-severe SNHL in the right ear. The left ear was not tested as previous testing had documented a profound SNHL in this ear. The audiogram of subject 2108 indicated a mild-to-moderately severe hearing loss in the right ear, that was mixed in the low frequencies and sensorineural in the mid and high frequencies. In the left ear, there was a severe low frequency mixed loss rising to a moderately severe high frequency SNHL. The age of each subject at the time of the audiogram is indicated in parenthesis. Arrows indicate no response at maximum audiometer output levels. Gray shaded area in the audiograms represents normal hearing range defined as air-conduction thresholds less than or equal to 15 dB HL for subjects less than 18 years, and less than or equal to 25 dB HL at 250 Hz to 8 kHz for adult subjects. y/o years old

**Fig. 3:**
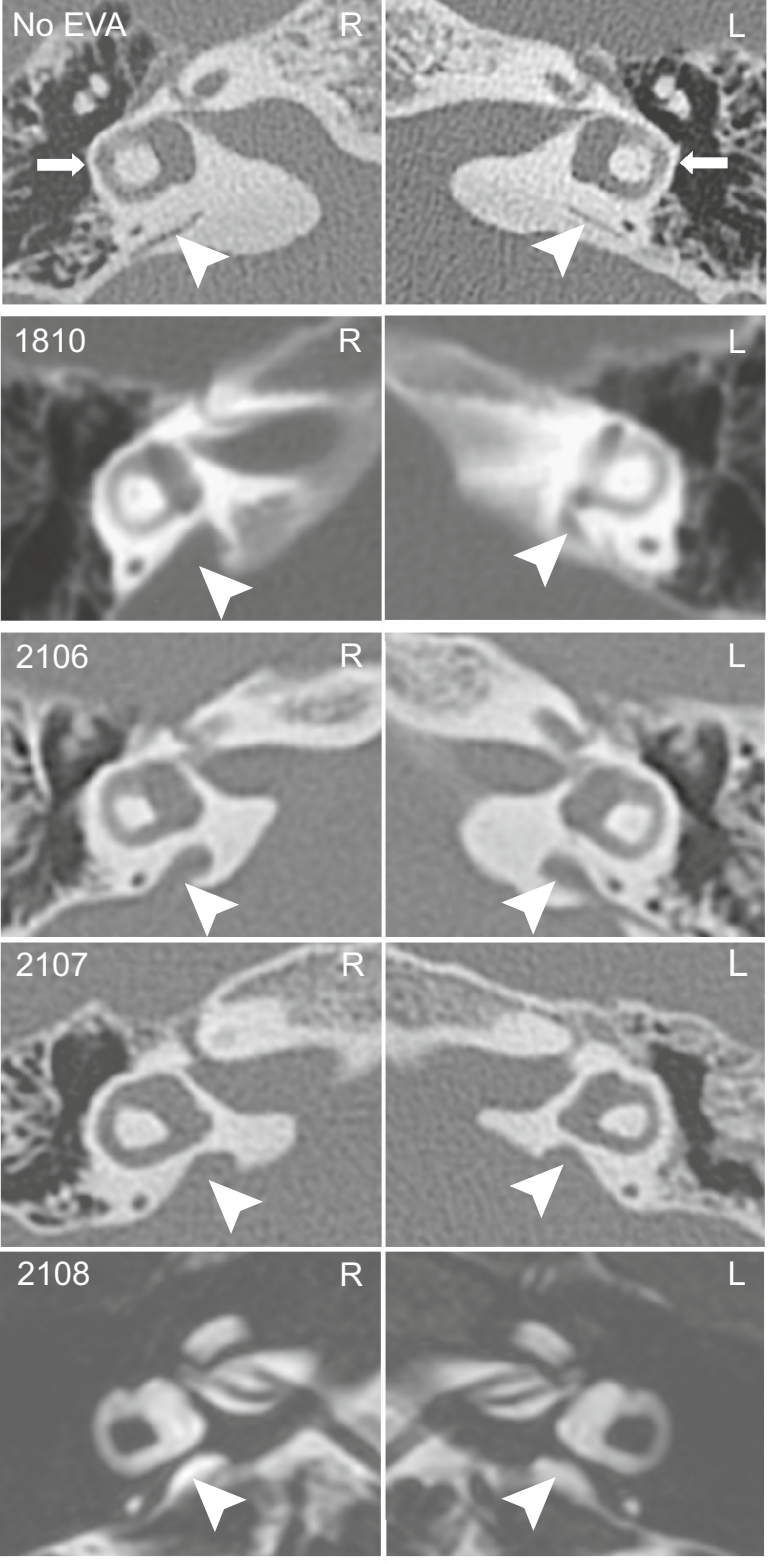
Radiological images of temporal bones of affected individuals with p.(Met1185Val) and p.(Gly1797Val) variants of *CHD7*. Montage of axial computed tomography (CT) (rows 1–4) and magnetic resonance (MR) (row 5) temporal bone imaging in a cohort subject with normal labyrinths and vestibular aqueducts (row 1), and from subjects with *CHD7* variant p.(Met1185Val) (row 2) and *CHD7* variant p.(Gly1797Ala) (rows 3–5). In row 1, normal anatomy is shown for reference, including the lateral semicircular canals (white arrows), which are often hypoplastic or absent in patients with CHARGE syndrome, and the normal vestibular aqueducts (white arrowheads) with a midpoint diameter less than 1 mm. For each patient (1810, 2106–2108), the lateral semicircular canals are well-formed, indistinguishable from normal (row 1, white arrow) and the vestibular aqueducts are enlarged (rows 2–5, white arrowheads) measuring 3 to 6 mm at the midpoint in each case. *R* right ear, *L* left ear

Subject 1810 had a fluctuating, asymmetric mixed hearing loss bilaterally. In the left ear, a slight-to-mild conductive hearing loss was limited to the low and mid frequencies. In the right ear, a moderate low frequency conductive loss and a moderate-to-severe, mid-to-high frequency mixed hearing loss that was primarily sensorineural was documented (Fig. 2). Subject 1810 used bilateral hearing aids that were first prescribed when he was approximately four and a half years old. No evidence of middle ear pathology was identified by CT imaging of the temporal bones (Fig. 3), audiologic immittance testing, or physical examination. His right and left vestibular aqueducts were enlarged (Fig. 3) with measured midpoint diameters of 6 and 3 mm, respectively. The lateral, posterior, and superior semicircular canals were morphologically normal bilaterally (Fig. 3). The remainder of his medical and developmental phenotype was significant for prenatal and antenatal ultrasound examinations demonstrating a right ureterocele and torturous right ureter requiring endoscopic repair of ureteral obstruction and reimplantation of the ureter. He had a duplication of the right kidney requiring nephrectomy at 15 months of age, a history of episodic nausea, vomiting, and torticollis, a constellation of signs and symptoms which is commonly observed in infants with EVA, presumably due to inner ear vestibular dysfunction. He was first noted to independently ambulate at 21 months of age. Ophthalmologic evaluation at 5 years of age reported normal retinas. He had an adenoidectomy at 5.5 years of age for an “airway issue” but no evidence of choanal atresia by physical examination or CT imaging of the skull base. MR imaging of the brain at 5 years of age revealed relative hypoplasia of the inferior vermis of the cerebellum with concomitant mild enlargement of the fourth ventricle. He was reported to have a short systolic murmur heard best at the left sternal border and thought to be innocent in nature. FISH hybridization in the long arm of chromosome 22 (22q11.2) was normal. Physical examination at 6 years of age (by A.J.G.) was notable for a narrow face with mandibular and malar hypoplasia, dysmorphic soft pinnae, and a high-arched palate with a normal uvula. He had single transverse palmar creases bilaterally. Both his mother (1812), father (1813), and sister (1811) had normal hearing sensitivity at all frequencies in both ears (Fig. 2). The father (1813), 41 years, reported a history of rare episodes of otitis media and limited noise exposure. His medical history included mild environmental allergies, mild myopia and surgery for removal of a vascular malformation from his left forearm. No lesions, masses or signs of syndromic hearing loss were detected by a physical examination focused on the head and neck.

#### Family 388

The variant c.5390G > C, p.(Gly1797Ala) was identified in monozygotic twin brothers (2107 and 2108) and their mother (2106), all with bilateral EVA, in family 388 (Table 1, Fig. 1B, C). This family of European descent was previously reported (Chattaraj et al. 2017; Muskett et al. 2016). p.(Gly1797Ala) has a REVEL score of 0.8989, a CADD score of 28.0 and an allele frequency of 0.00002. In silico analyses predicted it to be damaging (SIFT), probably damaging (PolyPhen), damaging (FATHMM-XF), and disease-causing (Mutation Taster). Glycine is conserved at this position among orthologs of CHD7 (Fig. 1D). This variant had previously been identified in a patient with CHARGE syndrome and in a patient with hypogonadotropic hypogonadism 5 (ClinVar VCV000263466.8 accessed June 20, 2022, classified as variant of uncertain significance). A variant thought to be pathogenic and affecting the same amino acid was identified as de novo in a patient with CHARGE syndrome with hearing loss p.(Gly1797Val) (Janssen et al. 2012). Another variant p.(Gly1797Arg) affecting this amino acid arose de novo in a patient with clinical features of CHARGE syndrome (Invitae, ClinVar RCV000705652.2, variant classified as likely pathogenic) and was also found in a patient with microphthalmia (p.(Gly1797Arg) and p.(Gly1233Ser)) (Patel et al. 2019). A second variant in *CHD7*, c.8950C > T, p.(Leu2984Phe), was identified in *trans* in the monozygotic twins 2107 and 2108, inherited from their father who does not have EVA. The Leu2984 residue is incompletely conserved among vertebrate orthologs (Fig. S2) and c.8950C > T has a very high maximum MAF in some populations (0.013 in Europeans of Finnish ancestry) (Table 1), arguing against its pathogenic potential.

Subjects 2106, 2107, and 2108 had bilateral hearing loss (Fig. 2). The hearing loss for subject 2016 was reported to be initially diagnosed when she was two-and-a-half years and progressed gradually. As a child, she had a history of chronic middle ear effusion that did not respond to antibiotics or several tube placements at ages ranging from 4 to 7 years. She reported experiencing tinnitus occasionally, worse in the left ear. She received a cochlear implant in her left ear at 35 years of age. The audiogram obtained when she was 41 years for her unimplanted right ear shows a severe to profound mixed hearing loss for the low frequencies and no measurable hearing at 1000 Hz and above (Fig. 2). Subject 2107 had hearing loss identified at two-and-a-half years of age. He had profound hearing loss in the left ear, and his right ear had fluctuating hearing loss which progressed to a moderate-to-severe SNHL at 6 years of age (Fig. 2). He wore hearing aids starting at 4 years of age and had bilateral cochlear implantation at 6 years of age. He experienced occasional vertigo but denied having experienced tinnitus. He had two episodes of otitis media and had a tube placed in the right tympanic membrane at 4 years of age. He had head trauma at 5 years of age, with subsequent nausea and vomiting, but no loss of consciousness or change in hearing status. Subject 2108 had hearing loss reportedly identified at 7 years of age. He experienced progressing and fluctuating hearing loss in both ears. At the time of his visit at NIH, when he was 8 years, his audiologic assessment revealed a sloping mild to moderately severe hearing loss in the right ear, that was mixed in the low frequencies, and SNHL in the high frequencies. In the left ear, he had a severe low frequency mixed loss rising to a moderately severe high frequency SNHL (Fig. 2). Immittance audiometry showed normal middle ear function bilaterally. He wore a hearing aid in the right ear and was in the process of being fitted with one for the left ear. His history was significant for one episode of hearing loss and vertigo with nausea, but no loss of consciousness, following a head trauma when he was 6 years. He had not experienced tinnitus or otitis media.

Subjects 2106, 2107, and 2108 all had findings consistent with bilateral EVA by CT (subjects 2106 and 2107) or MRI (subject 2108) (Fig. 3) with measured midpoint diameters of 3 and 3 mm (right and left) for subject 2106, 4 and 4 mm for subject 2107, and 4 and 5 mm for subject 2108. Semicircular canal morphology was normal bilaterally for subjects 2106 and 2108 (Fig. 3). The lateral semicircular canals of 2107 were slightly dysmorphic with reduced size of the bony island bilaterally. The cochleae of subject 2108 also had incomplete partitions bilaterally (not shown). Medical-developmental histories were significant for subject 2106 who anamnestically self-reported an asymptomatic viral infection that caused “retinal tears” in her left eye at the age of 15 years. She did not recall any symptoms or signs that led to the diagnosis of retinal abnormalities. She had a limited non-dilated funduscopic examination (by A.J.G.) that could not detect any retinal abnormalities at the age of 41 years. Medical records were not helpful and dilated funduscopy could not be performed to confirm the existence of a retinal abnormality, such as a subclinical coloboma. Ultrasonography of the renal and collecting systems was normal for all three subjects. Thyroid ultrasonography detected a few small (1 to 2 mm) parenchymal cysts in subjects 2107 and 2108, but perchlorate discharge and thyroid serologic test results were within normal limits for all three subjects.

### Structural models of wild-type and variant CHD7 proteins

**Fig. 4:**
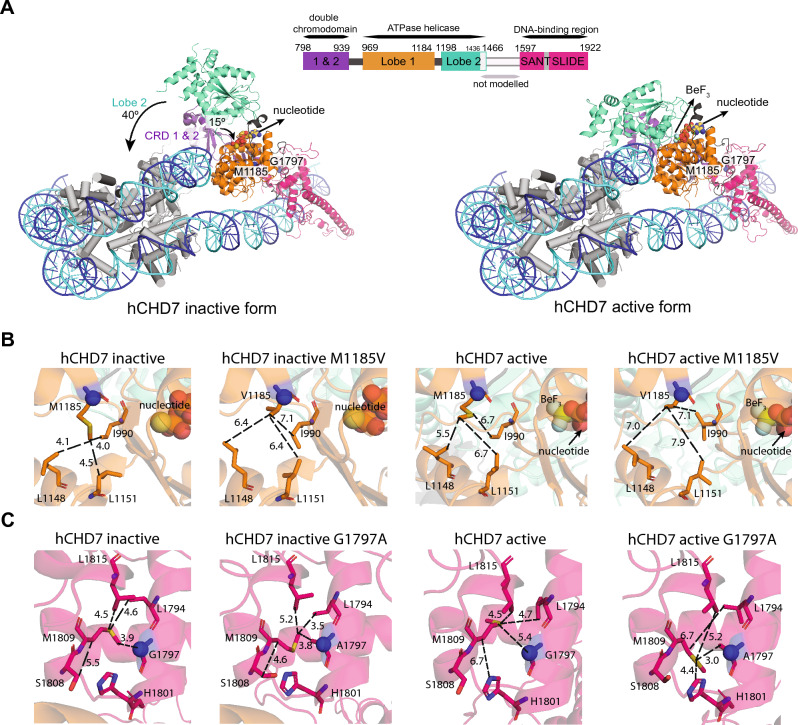
Structural models of human CHD7 in active and inactive states. **A** Structural models based upon the alignment shown in Fig. S3 of CHD7 containing two chromodomains (CRD 1 and 2, purple), an ATPase motor (lobe 1 in orange and lobe 2 in green) and a SANT–SLIDE domain (pink) that binds DNA. Domains are colored as in Farnung et al. (Farnung et al. 2017). The coordinates of the histone complex (shown in gray cylinders) and the double strand DNA (blue) in CHD7 active form were obtained for purpose of visual display, after structural superimposition of the active template structure onto the model obtained. For the inactive model, the coordinates of the histone complex and the double strand DNA were obtained after structural superimposition of lobe 1 of the ATPase motor and SANT–SLIDE domains of the active template onto the inactive model. C-alpha atoms of residues Met1185 and Gly1797 are shown as blue spheres, while the atoms forming the nucleotides are shown as yellow (C), red (O), orange (P), blue (N) spheres. BeF3 is shown as cyan spheres (right model). Structural changes in CHD7 between the active and inactive states are indicated with arrows: the 40° rotation of lobe 2 in the ATPase motor and the 15° rotation of the chromodomains with respect to the DNA. **B**, **C** Close-up views of the structural models of inactive and active states of wild-type CHD7, and with the substitutions p.(Met1185Val) (**B**) and p.(Gly1797Ala) (**C**). The residues at positions 1185 and 1797 as well as those within 6 Å of the variant site are shown as sticks. The interacting networks involving Met1185 and Gly1797, Val1185 and Ala1797 are indicated as dashed lines. The corresponding C-alpha atom of each of these residues is shown as a blue sphere

To gain insight into the structural effects of these substitutions, we used computational methods to generate structural models of wild-type and mutated human CHD7 in its active and inactive states. CHD1 from *Saccharomyces cerevisiae* in active (PDB id: 5og9) and inactive (PDB id: 3mwy) states (Farnung et al. 2017; Hauk et al. 2010) were used as templates. The initial alignment of CHD7 and CHD1 covered the two chromodomains, the ATPase motor and the SANT–SLIDE domains with a sequence identity of 35%. This alignment was refined using an iterative procedure. The refined sequence alignment shows an overall correspondence between secondary structural elements (Fig. S3B) indicating that both proteins, CHD7 and CHD1, share the same fold. The structural CHD7 models obtained have Met1185 located in lobe 1 of the ATPase motor participating in a hydrophobic interaction network that stabilizes two helices and a beta-strand, with the latter close to the nucleotide-binding site (Fig. 4A, B). The residues in this region are more tightly packed when CHD7 is inactive than when it is active. The substitution p.(Met1185Val) introduces a shorter side chain in this position, completely abolishing the hydrophobic interacting network, since it leads to distances between side-chains larger than 6 Å, when the typical range for a hydrophobic interaction is 3–6 Å, in the inactive form, as well as the already weak network present in the active state. This computational analysis suggests that p.(Met1185Val) destabilizes the local fold of the protein in a region that is close to the nucleotide binding site and that this change might be more disruptive to the inactive form than the active form. While the local destabilization of the fold might not abolish CHD7 function, the proximity of the variant to the nucleotide binding site may have a negative impact on CHD7 function.

Gly1797 is part of the SANT–SLIDE domain and is located between two alpha-helices in a tightly packed region (Fig. 4A, C), where a central Met1809 interacts with most of the residues in this zone and is located 3.9 Å from Gly1797 C-alpha in the inactive form, in comparison with 5.4 Å in the active form. The substitution p.(Gly1797Ala) introduces a methyl group in this tightly packed region inducing steric clashes between Met1809 and nearby residues, which indicates destabilization of the local fold and possible negative impact on CHD7 activity. The variant p.(Gly1797Ala) has an especially negative impact in the active form positioning Met1809 only 3 Å from Ala1797 and destabilizing Met1809 interactions with Leu1815 and Leu1794. These results show a negative impact of p.(Gly1797Ala) variant in the local fold of the protein, especially the active form.

The rearrangements observed for p.(Met1185Val) and p.(Gly1797Ala) were observed not only in the best model for each variant, but also for the best five models of each run.

### *Chd7* expression in developing mouse endolymphatic duct and sac

**Fig. 5:**
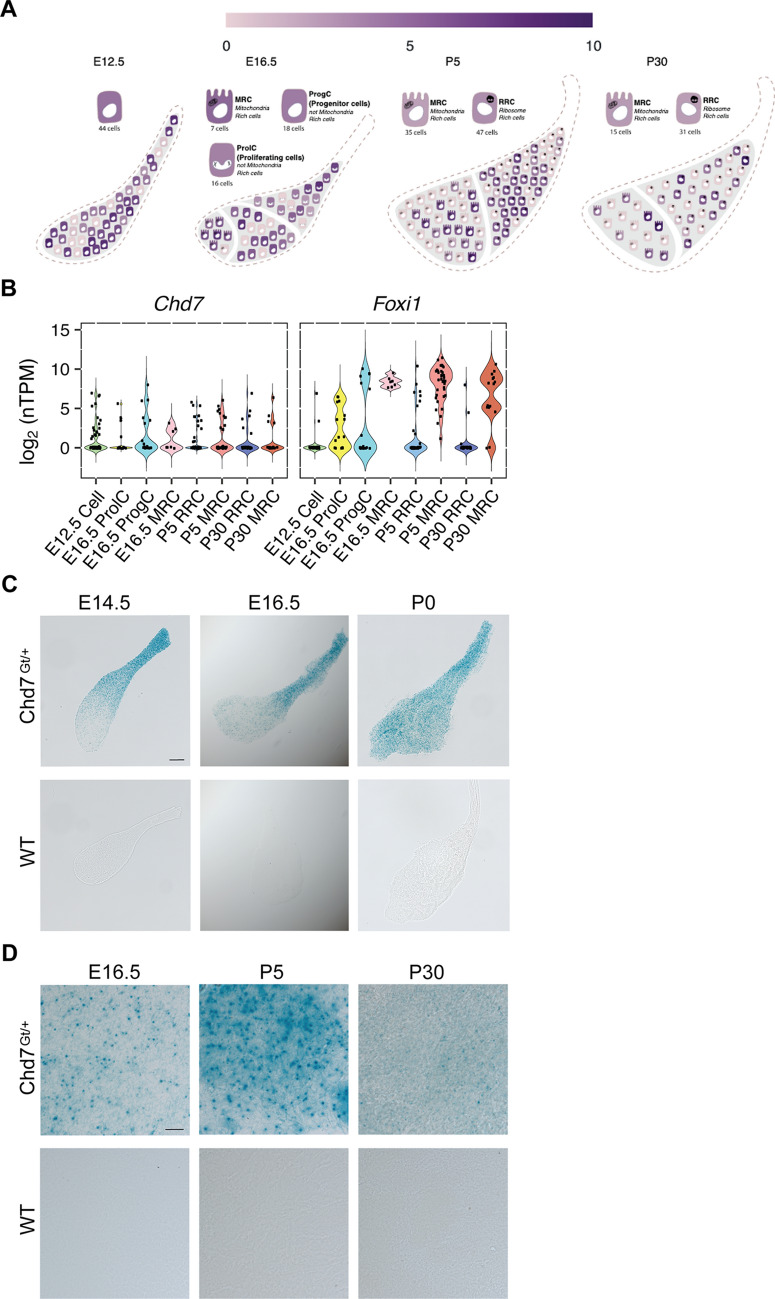
*Chd7* expression in the developing mouse endolymphatic sac and duct. **A**, **B** Single cell expression data (scRNA-seq (Honda et al. 2017)) showing *Chd7* expression in the different cell types of the developing mouse endolymphatic sac at E12.5, E16.5, P5 and P30 visualized in the gEAR portal (https://umgear.org) (**A**). Violin plots of *Chd7* and *Foxi1* expression in the cells of the endolymphatic sac and their precursor cells (**B**). While the transcription factor *Foxi1* is expressed preferentially in MRCs and their precursor cells, *Chd7* is expressed in all cell types of the endolymphatic sac. Each cell group is labelled based on expression patterns of canonical cell markers and results of gene ontology enrichment analysis. ProlC, proliferating cells (representative differentially expressed genes are *Mki67, Birc5, Tpx2*); ProgC, progenitor cells (*Lmx1a, Bmp3, Col11a1*); MRC, mitochondria-rich cells (*Slc26a4, Foxi1, Atp6v0a4, Atp6v1b1*); RRC, ribosome-rich cells (*Agt, Cav1, Clu, Dmkn*); Values: TPM transcript per million. **C**, **D** Developmental expression pattern of *Chd7* in the endolymphatic sac and duct of *Chd7*^*Gt*/+^ mice revealed by X-gal staining. Low magnification view of whole-mount X-gal staining of *Chd7*^*Gt*^^/+^ endolymphatic sac and duct preparations show *Chd7* expression at E14.5, E16.5 and P0 (**C**). Close-up views of the endolymphatic sac at higher magnification at E16.5, P5 and P30 (**D**). X-gal staining (blue dots) was detected in the different regions of the endolymphatic sac and along the endolymphatic duct at E14.5, E16.5, P0. Staining seemed to be more intense in the endolymphatic duct at E14.5, E16.5, as compared to the rest of the endolymphatic sac, whereas the staining seemed more homogenous at P0. At P5 and P30, staining was still detected in the endolymphatic sac. No staining was detected in endolymphatic sac and duct tissue from wild-type (WT) littermates processed in parallel. Three to four litters were studied for each datapoint. Whenever possible, animals of both sexes were studied and observed to have no obvious differences. Scale bars: 100 μm (**C**), 20 μm (**D**)

The observed EVA malformations could be the direct consequence of an enlarged endolymphatic sac and duct due to dysfunctional CHD7 in this tissue, or due to other secondary effects of the role of CHD7 in inner ear formation. We thus aimed at studying the expression of CHD7 in the endolymphatic sac and duct using mouse as a model. *Chd7* transcripts were detected in cells of the mouse endolymphatic sac epithelium at E12.5, E16.5, P5 and P30 (Fig. 5A, B) in published single-cell RNA-seq data (Honda et al. 2017). In contrast to *Foxi1*, a transcription factor preferentially expressed in a subtype of endolymphatic sac cells, the mitochondria-rich cells and their precursor cells, *Chd7* expression was detected in all cell types of the endolymphatic sac epithelium at all ages tested (Fig. 5B).

To further examine the temporospatial expression of *Chd7* in the developing endolymphatic sac and duct, we used mice segregating a reporter allele, *Chd7*^*Gt*^. In *Chd7*^*Gt/*+^ mice, β-galactosidase activity visualized by X-gal staining is expected to mimic the endogenous expression pattern of *Chd7* mRNA (Hurd et al. 2007). We observed β-galactosidase activity in the endolymphatic sac and duct at all ages tested: E14.5 (endolymphatic sacs from 5 males (M) and 4 females (F), E16.5 (6 M and 3 F), P0 (4 M and 3 F), P5 (1 M and 2 F) and P30 (2 M, 1 F) (Fig. 5C, D). No staining was visible in the endolymphatic sac and duct of wild-type littermates processed in the same conditions (E14.5 (endolymphatic sacs from 5 M, 1 F), E16.5 (1 M and 2 F), P0 (5 M and 3 F), P5 (3 M and 1 F) and P30 (1 M, 3 F)). Limited data are available at postnatal ages, because we had difficulty breeding these mice to adulthood. Intriguingly, X-gal staining seemed more intense in the endolymphatic duct than in the endolymphatic sac at E14.5 and E16.5, whereas the staining was more homogenous at P0. No obvious difference between sex was identified.

**Fig. 6:**
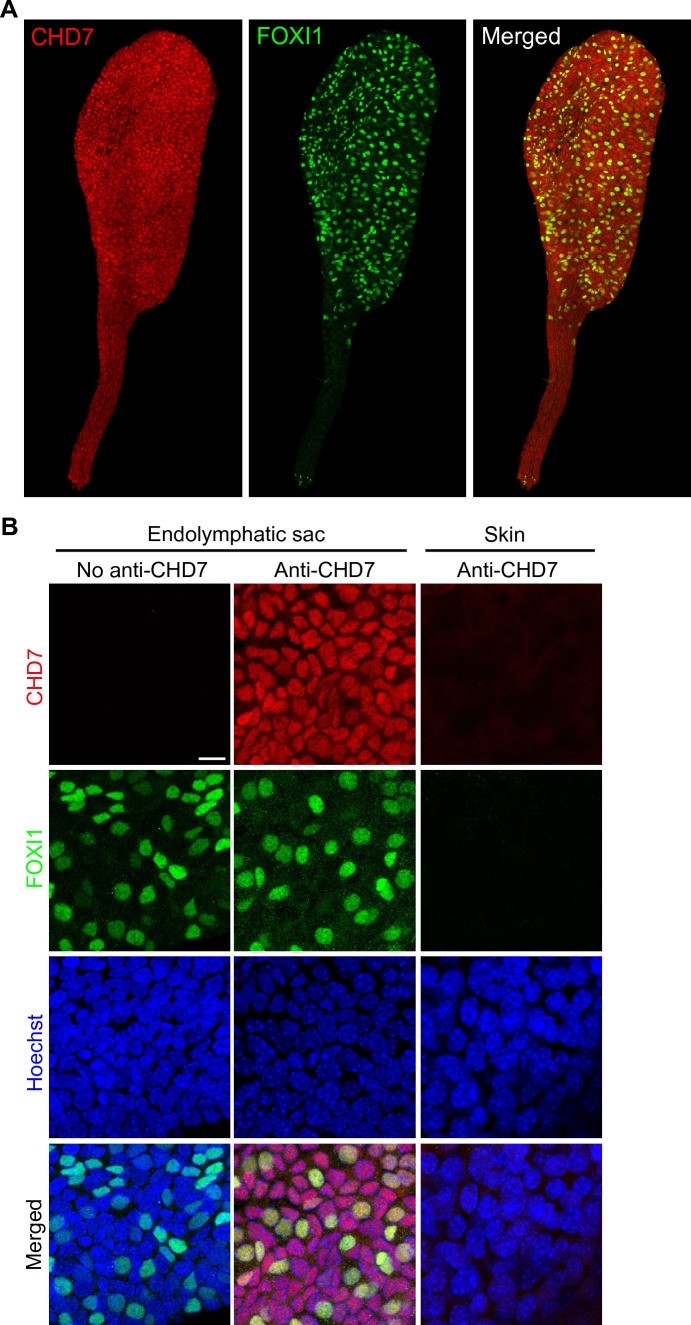
Immunolocalization of CHD7 in the developing mouse endolymphatic sac and duct. Confocal microscopy analysis of whole-mount endolymphatic sac and duct preparations from E16.5 mice labelled with antibodies directed against CHD7 (red) and FOXI1 (a mitochondria-rich cell marker, green). Hoechst 33342 (blue) was used to label cell nuclei. Experiments were conducted with two different antibodies directed against CHD7 N-terminal region, with similar results. Here are shown the results with Alexa Fluor 594 conjugated anti-CHD7 from Novus. Maximal intensity projection images at E16.5. **A** Low magnification images of the endolymphatic sac and duct. **B** Higher magnification images of the open endolymphatic sac. No labeling was detected in the absence of primary antibodies, or in skin tissue used as a negative control. CHD7 was detected in the nuclei of the cells all along the endolymphatic duct and in both mitochondria-rich cells and ribosome-rich cells in the endolymphatic sac. Scale bars: 100 μm (**A**), 10 μm (**B**)

CHD7 immunoreactivity was detected in the developing mouse endolymphatic sac and duct at E16.5 (Figs. 6A, B, S4). Two independent antibodies gave similar results. Co-labeling with Hoechst 33342 and anti-FOXI1 antibodies showed that CHD7 immunoreactivity was enriched in the nuclei of the cells of the endolymphatic sac and duct. It was present in all cells regardless of whether they showed FOXI1 immunoreactivity (Figs. 6A, B, S4). The specificity of CHD7 labeling in the endolymphatic sac and duct was supported by the lack of labeling detectable in skin tissue processed in similar conditions (Figs. 6B, S4). Similar labeling was obtained for the endolymphatic sac from both males and females (for each antibody, the labelings of two animals of each sex from two different litters were compared).

## Discussion

This study evaluated the contribution of *CHD7* variant alleles to the etiology of EVA and associated SNHL in families without pathogenic variants in *SLC26A4*. Two candidate causal variants of *CHD7*, p.(Met1185Val) and p.(Gly1797Ala), were each identified in one family. p.(Met1185Val) is a variant of uncertain significance and (Gly1797Ala) is a likely pathogenic variant according to ACMG/AMP guidelines for the interpretation of sequence variants in hearing loss genes (Oza et al. 2018). These variants are rare and their potential pathogenicity is supported by their strong conservation across species, in silico analyses, and our computational modeling showing the destabilizing effects of these variants on the local fold of the protein. In addition, we identified potential manifestations of CHARGE in the individuals with EVA and SNHL who carried these variants of *CHD7*. One subject (1810) from one family had genitourinary malformations consistent with CHARGE. Another adult subject (2106) from the other family self-reported a retinal finding that might have represented a subclinical coloboma, which unfortunately, could not be confirmed. Both the lack of phenotype in the father of individual 1810 carrier of the same *CHD7* variant as his son, and the variable phenotype reported in carriers of the variant p.(Gly1797Ala), which varies from CHARGE syndrome to hypogonadotropic hypogonadism 5 and, here, SNHL associated with EVA, is not unexpected. Indeed the variable penetrance and expressivity of the signs associated with *CHD7* variants both within and between families has been well-documented, in particular when missense variants are involved and a milder phenotype of CHARGE syndrome is seen (Delahaye et al. 2007; Jongmans et al. 2008, 2009; Kim et al. 2008). The lack of clinical follow-up data is a limitation of our study, especially since many of our subjects were ascertained at young ages before the expected onset of puberty. From the available information, we also cannot conclude whether the variant p.(Gly1797Ala) was maternally or paternally inherited for the individual 2106, or even present in a mosaic state in one of her parents, as germline mosaicism has previously been described in several cases for variants of *CHD7* (Jongmans et al. 2008; Lalani et al. 2006; Pauli et al. 2009).

None of the four subjects with EVA and a likely causal variant of *CHD7* had major semicircular canal malformations. This is noteworthy, since about 95% of patients with *CHD7* pathogenic missense variants have semicircular canal anomalies (Bergman et al. 2012b). Conversely, all four of our study subjects had bilateral EVA, whereas EVA has been reported to be present in only 10% of CHARGE patients, most frequently as unilateral EVA (Abadie et al. 2000; Bedeschi et al. 2020; Hoch et al. 2017; Vesseur et al. 2016). Although their temporal bone histopathology revealed an enlargement of the most proximal part of the endolymphatic duct, a recent report by da Costa et al. concluded that none of six study subjects with CHARGE syndrome had EVA (da Costa Monsanto et al. 2022). Intriguingly, *Pax2-Cre;Chd7*^*Gt/flox*^ mice exhibit an enlarged endolymphatic duct (Balendran et al. 2021). The mechanisms underlying the difference in the expressivity and penetrance of EVA and semicircular canal malformations associated with pathogenic variants of *CHD7* are unknown. Possible explanations include a variant at another locus encoding a CHD7-interacting protein (whether physically interacting or involved in a genetic/synergistic interaction) required for development of the endolymphatic sac and duct, as previously described in Kallmann syndrome and hypogonadotropic hypogonadism (Marcos et al. 2014). Another possibility is intrinsic differences in the effects of different *CHD7* variants. The missense variants identified in this study were predicted to affect relative stabilities of local folds of the CHD7 protein, but could also influence binding to cell- and tissue-specific proteins which interact with CHD7, a key regulator of inner ear development expressed in almost all cells and with potential roles at different times during the development of the inner ear. Our study indicates that *Chd7* mRNA is present in the developing endolymphatic sac and duct, supporting the hypothesis of its involvement in a developmental pathway in addition to or different from that which underlies semicircular canal development. We observed immunoreactivity with anti-CHD7 antibodies but were unable to definitively confirm the presence of CHD7 protein in the endolymphatic sac and duct due to the lack of control tissue without *Chd7* expression. In *Chd7*^*Gt/*+^ mice, a strong X-Gal staining was found at E14.5 and E16.5 in the endolymphatic duct as compared to the endolymphatic sac itself. Such a difference was not seen by immunohistochemistry at E16.5. This could be due to technical issues, or differences in the regulation of *Chd7* mRNA and the corresponding protein. X-Gal staining seemed more homogenous at P0. Expression of *Chd7* in the mouse endolymphatic sac persisted at least until P30 in the *Chd7*^*Gt/*+^ mice. Our immunolabeling experiments did not detect CHD7 in the adult human endolymphatic sac, although it was detected in spiral ganglion neurons (not shown). The possible expression of CHD7 in the human endolymphatic sac was not tested at embryonic age.

The presence of pathogenic variants in the genes *EYA1, SIX1, SIX5*, *PAX3*, *MITF*, *SNAI2*, *SOX10*, *EDN3*, and *EDNRB* was also investigated in this cohort, but did not detect any potential pathogenic variant in these genes associated with BOR, BO and Waardenburg syndromes. It remains possible that copy number variants or large chromosomal rearrangements could have been missed by our approach based on exome sequencing. However, this seems unlikely for *CHD7,* since large deletions and other variants leading to absent or truncated proteins have been described to result, in general, in a more severe CHARGE syndrome phenotype than missense variants (Bergman et al. 2012b; Janssen et al. 2012; Kim et al. 2008).

*CHD7* variants should be included in molecular testing for EVA, especially in those patients who do not carry *SLC26A4* pathogenic variants. Clinical evaluations should be alert to the possibility of CHARGE manifestations. Contrary to EVA associated with *SLC26A4*, which is transmitted in a recessive manner (Chattaraj et al. 2017), in patients with *CHD7* variants, the inheritance is dominant but the EVA phenotype is incompletely penetrant, and may appear sporadically. Future studies are needed to test our hypothesis that some *CHD7* variants can cause EVA with an atypical CHARGE phenotype or even a nonsyndromic form of EVA. The study of additional patients with EVA and *CHD7* variants will allow further exploration of the basis for the differences in phenotype in our patients versus those with CHARGE and other variants of *CHD7*.

### Supplementary Information

Below is the link to the electronic supplementary material.

Segregation of EVA and hearing loss (black symbol) in families 258, 281, 234, 284 and 347. Individual 2004 in family 347, was an adult with hearing thresholds within normal limits for all tested frequencies in the right ear, whereas his left ear demonstrated a high frequency SNHL. This phenotype is not consistent with the presence of EVA (striped symbol). “+” symbolizes wild-type alleles. The genotype is indicated for all the gDNA available. The variant p.(Gly744Ser) had been reported in a patient of North African ancestry presenting with Kallmann syndrome and hearing loss, and in another patient of African ancestry presenting with an atypical form of CHARGE syndrome (atypical eyelid coloboma, hearing loss, severe developmental delay, ventricular septal defect, short stature, and abnormal facies, limb anomaly, primary hypoparathyroidism and interrupted pubertal development) (Jain et al. 2011; Marcos et al. 2014). Since the allele frequency of p.(Gly744Ser) is 0.0153 among Africans, these reported genotypic-phenotypic associations were likely coincidental. Although family 281 self-reported Caucasian ancestry, subject 1817 carries the same linked variants flanking p.(Gly744Ser) as the African patient with this variant (Jain et al. 2011). The high frequency of this variant in African populations renders it unlikely to be a pathogenic, however structural or intronic pathogenic variant in *cis* might have been missed.Supplementary file1 (PDF 396 KB)

Conservation of the residues affected by CHD7 missense substitutions across species.Conservation across vertebrate and invertebrate (*Drosophila melanogaster*, *Caenorhabditis elegans*) species of the residues affected by missense substitutions of CHD7. The percentage of identity is reflected by the blue background (the darker the highest identity). Sequences obtained through Uniprot website were analyzed in Jalview. Alignment was performed using ClustalW. Protein sequences identifiers for CHD7 orthologues are Q9P2D1 for *Homo sapiens*, F6PP91 for *Macaca mulatta*, F1PWD8 for Canis familiaris, G3UE09 for *Loxodonta africana*, F7G444 for *Monodelphis domestica*, A2AJK6 for *Mus musculus*, A0A452E916 for *Capra hircus*, A0A674K692 for *Terrapene carolina triunguis*, Q06A37 for *Gallus gallus*, U3JST3 for *Ficedula albicollis*, A0A1L8FT46 for *Xenopus laevis*, F1QGL1 for *Danio rerio*, M9NEL3 for *Drosophila melanogaster*, O61845 for *Caenorhabditis elegans*. Supplementary file2 (AI 558 KB)

Inactive template used during the modelling production run. **A** The inactive template used to model human CHD7 inactive state was obtained after structural superimposition of lobe 1 in the ATPase motor of structures 5o9g and 3mwy. The final template contained chromodomains 1 and 2, lobe 1 and 2 of the ATPase motor from the inactive structure and the SANT-SLIDE domains from the active structure after superimposition. **B** Amino acid sequence alignment between CHD7 and the templates in active (PDB id: 5o9g) and inactive (PDB id: 3mwy) states used during the modelling procedure. The secondary structure prediction (SSP) averaged over the CHD7 profile from Hhpred server (Hildebrand et al. 2009; Zimmermann et al. 2018) as well as the secondary structure (SS) for both templates are shown above CHD7 and bellow inactive sequences as gray (coil), blue (helix) and red (beta-strand) bars. The two amino acids Met1185 and Gly1797 affected by the variants found in families 276 and 388 are indicated by green spheres above the SSP. Residues shown in the model are highlighted according to their properties: neutral in white, aromatic in orange, polar in green, basic in blue, acidic in red, and Gly and Pro in pink. Supplementary file3 (AI 24381 KB)

Confocal analysis of whole-mount endolymphatic sac preparations from E16.5 mice labelled with antibodies directed against CHD7 (red) and FOXI1 (a mitochondria-rich cell marker, green). Hoechst 33342 (blue) was used to label cell nuclei. Experiments were conducted with two different antibodies directed against CHD7 amino-terminal region, with similar results. The results shown here were obtained with anti-CHD7 from Cell Signaling. Maximal intensity projection images are presented. No staining was detected in the absence of primary antibody, or in skin tissue incubated with anti-CHD7 antibodies and processed in parallel. CHD7 was detected in the nuclei of both mitochondria-rich cells and ribosome-rich cells in the endolymphatic sac. Scale bar: 10 μm. Supplementary file4 (AI 10806 KB)

Supplementary file5 (DOCX 22 KB)

## Data Availability

The variants NM_017780.4: c.3553A > G, p.(Met1185Val) and c.5390G > C, p.(Gly1797Ala), associated with hearing loss and EVA, have been submitted to the ClinVar public database (Landrum et al. 2016) under the references SCV002817423 and SCV002817424. All data relevant to the study are included in the article or uploaded as online Supplementary Information. For further information, contact the corresponding author.
