# Supplementary material for: CHD7 variants associated with hearing loss and enlargement of the vestibular aqueduct
Source: Hum Genet. 2023 Sep 5;142(10):1499–517. doi: 10.1007/s00439-023-02581-x (PMC10511616; doi:10.1007/s00439-023-02581-x)
Supplement: Supplementary file 1 — Segregation of EVA and hearing loss (black symbol) in families 258, 281, 234, 284 and 347. Individual 2004 in family 347, was an adult with hearing thresholds within normal limits for all tested frequencies in the right ear, whereas his left ear demonstrated a high frequency SNHL. This phenotype is not consistent with the presence of EVA (striped symbol). “+” symbolizes wild-type alleles. The genotype is indicated for all the gDNA available. The variant p.(Gly744Ser) had been reported in a patient of North African ancestry presenting with Kallmann syndrome and hearing loss, and in another patient of African ancestry presenting with an atypical form of CHARGE syndrome (atypical eyelid coloboma, hearing loss, severe developmental delay, ventricular septal defect, short stature, and abnormal facies, limb anomaly, primary hypoparathyroidism and interrupted pubertal development) (Jain et al. 2011; Marcos et al. 2014). Since the allele frequency of p.(Gly744Ser) is 0.0153 among Africans, these reported genotypic-phenotypic associations were likely coincidental. Although family 281 self-reported Caucasian ancestry, subject 1817 carries the same linked variants flanking p.(Gly744Ser) as the African patient with this variant (Jain et al. 2011). The high frequency of this variant in African populations renders it unlikely to be a pathogenic, however structural or intronic pathogenic variant in cis might have been missed.Supplementary file1 (PDF 396 KB) [file 439_2023_2581_MOESM1_ESM.pdf]

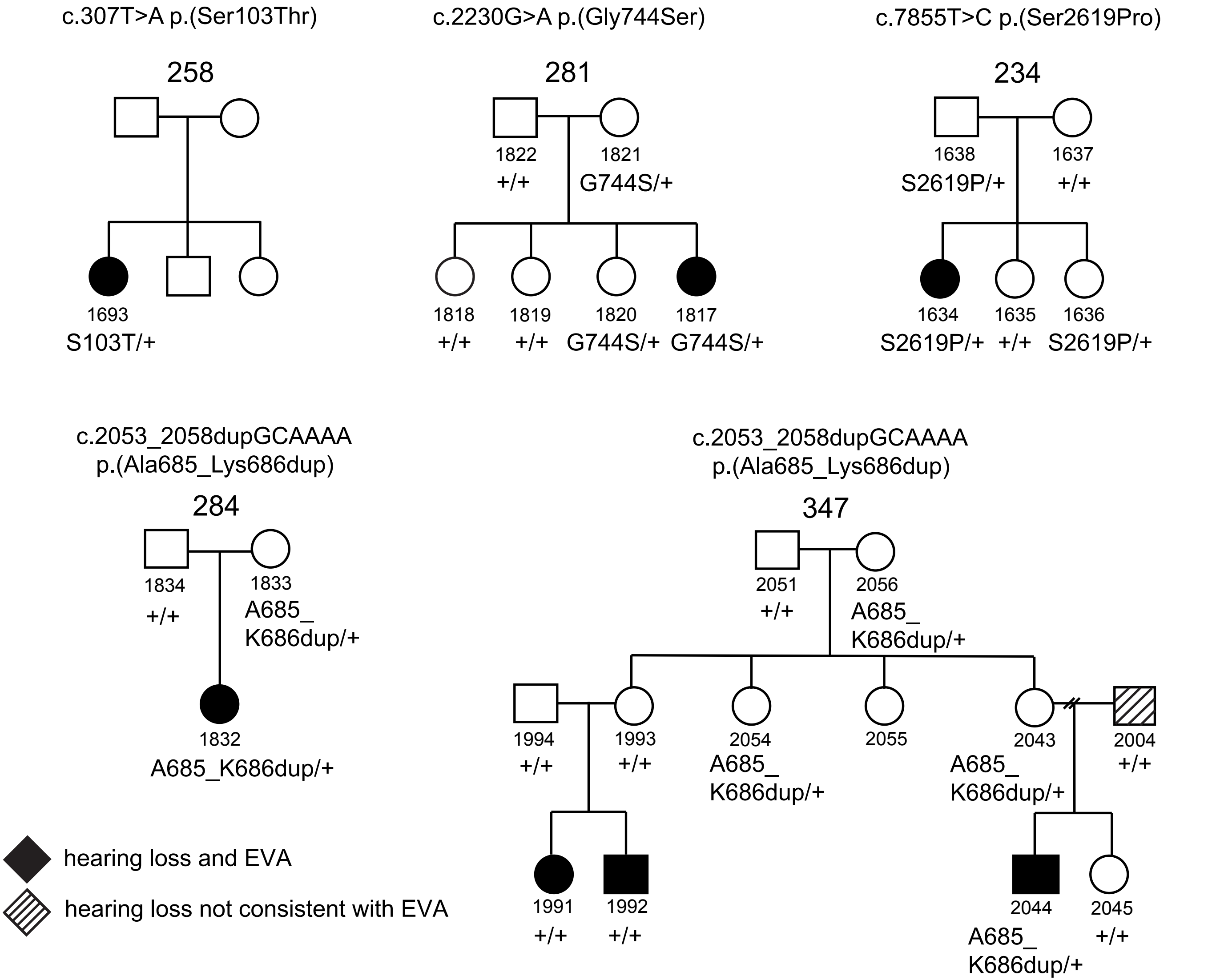
c.307T>A p.(Ser103Thr)
258
1693
S103T/+
c.2230G>A p.(Gly744Ser)
281
1822
+/+
1821
G744S/+
1818
+/+
1819
+/+
1820
G744S/+
1817
G744S/+
c.7855T>C p.(Ser2619Pro)
234
1638
S2619P/+
1637
+/+
1634
S2619P/+
1635
+/+
1636
S2619P/+
c.2053_2058dupGCAAAA
p.(Ala685_Lys686dup)
284
1834
+/+
1833
A685_
K686dup/+
1832
A685_K686dup/+
c.2053_2058dupGCAAAA
p.(Ala685_Lys686dup)
347
2051
+/+
2056
A685_
K686dup/+
1994
+/+
1993
+/+
2054
A685_
K686dup/+
2055
2043
A685_
K686dup/+
2004
+/+
1991
+/+
1992
+/+
2044
A685_
K686dup/+
2045
+/+
hearing loss and EVA
hearing loss not consistent with EVA
