# Supplementary material for: CHD7 variants associated with hearing loss and enlargement of the vestibular aqueduct
Source: Hum Genet. 2023 Sep 5;142(10):1499–517. doi: 10.1007/s00439-023-02581-x (PMC10511616; doi:10.1007/s00439-023-02581-x)
Supplement: Supplementary file 5 — Supplementary file5 (DOCX 22 KB) [file 439_2023_2581_MOESM5_ESM.docx]

**Table S1. Primers used to amplify and confirm by Sanger sequencing the variants identified by exome sequencing in *CHD7* and to analyze *CHD7* haplotype in family 281.**

| Primer name including exon targeted | Primer sequence 5’ to 3’ | Targeted region on Chr.8  (hg. 38) | Annealing temperature  (^o^ C) | Amplicon size (bp) |
| --- | --- | --- | --- | --- |
| CHD7_2a_F^†^ | TTCAGGTACCAGACCAGATACG | 60741881-  60742464 | 60 | 584 |
| CHD7_2a_R^†^ | TACGGACTGATTCATTGGAGT |  |  |  |
| CHD7_2b_F^†^ | ACCAGATACGAGCCCCCTAC | 60741893-  60742484 | 60 | 592 |
| CHD7_2b_R^†^ | ACAGCATTGGGGTATCTTGG |  |  |  |
| CHD7_2c_F^†^ | GGCAGTATTCTCGATATCCTTACAG | 60742355-  60742919 | 60 | 565 |
| CHD7_2c_R^†^ | ATCAGTCGTTCCTGGATTGC |  |  |  |
| CHD7_2d_F^†^ | ATGCAGCAGTCTCGTCCATT | 60742795  60743268 | 60 | 474 |
| CHD7_2d_R^†^ | ACAGGGAGATTGATGCCTGA |  |  |  |
| CHD7_2a1F^*^ | AAATACAGCTCAGCGAGGGA | 60741118 |  |  |
| CHD7_2a1R^*^ | CCTCTTGGCCTTGGGAAAAC | 60742087 |  |  |
| CHD7_2a2F^*^ | TGCTTGCTTTCTAAGTAACCGA | 60741037 |  |  |
| CHD7_2a2R^*^ | TGCCATATAGCTGCCCATCT | 60742014 |  |  |
| CHD7_3_F^†^ | GAAACATCAGCCACTAACTTTCA | 60780917-  60781515 | 55 | 599 |
| CHD7_3_R^†^ | CCCCTCATTTCATAGGCTGTA |  |  |  |
| CHD7_4F^†#^ | GCCAATATGTATGGATTTATCAGTTG | 60794866-  60795217 | 60; 61^#^ | 352 |
| CHD7_4R^†#^ | CAAGATAGGGGAGGTCTTGTG |  |  |  |
| CHD7_15_F^†^ | CACTGGGCTTTGAAAAATGAA | 60830197-  60830665 | 58 | 469 |
| CHD7_15_R^†^ | CACCATGAAATCCCCAGTCT |  |  |  |
| CHD7_exon15seq1^*^ | GGATGTTTAATGAATGAGAT | 60830221 |  |  |
| CHD7_exon15seq2^*^ | TCCTGATGTTTCATGTTA | 60830243 |  |  |
| CHD7_exon15seq3^*^ | TATGTTTTAACTTGCAAG | 60830264 |  |  |
| CHD7_exon15seq4^*^ | ACTAGTATTTACTTAAGG | 60830293 |  |  |
| CHD7_25_F^†^ | CCCACCATGCTCAGATGTTT | 60848938-  60849275 | 58 | 338 |
| CHD7_25_R^†^ | GCCAAGAGTCCTTTGGAACT |  |  |  |
| CHD7_36_F^†^ | TCTGACAGTTCTCTTTGGCATT | 60862114-  60862408 | 60 | 295 |
| CHD7_36_R^†^ | GAAGGCAGGATAAAACACTTCC |  |  |  |
| CHD7_38a_F^†^ | ATTTGGAATGGCAGGTTCAC | 60864922-  60865205 | 60 | 284 |
| CHD7_38a_R^†^ | TCAGGTCCATTCCAGCAAAC |  |  |  |
| CHD7_38b_F^†^ | GAAGAGGAAGAAGGCCCAAA | 60865086-  60865675 | 60 | 590 |
| CHD7_38b_R^†^ | TCCTGGAGGTAGAAACATGG |  |  |  |
| CHD7_38c_F^†^ | TGACTCTGCGAATGGATCTG | 60865540-  60866012 | 60 | 473 |
| CHD7_38c_R^†^ | TGAACATTAACTGTGAGTGTAAACAG |  |  |  |
| CHD7_1817_1F^#^ | AAAGCTGACACATTTTGATCACT | 60741624-60742217 | 61 | 594 |
| CHD7_1817_1R^#^ | GTAGAGGGGTGGTGATGGAAC |  |  |  |
| CHD7_1817_2F^#^ | AGCTGGGAGAGAGGCTTAAG | 60852943-60853470 | 61 | 528 |
| CHD7_1817_2R^#^ | CGTCATCCTCCAGCTTTTCC |  |  |  |
| CHD7_1817_34F^#^ | AAGCCAGCCCATATAGCAGT | 60856378-60857007 | 61 | 630 |
| CHD7_1817_34R^#^ | GGAGGAAGCTGGCTTTCATA |  |  |  |

*Sequencing primers designed to get bi-directional chromatograms. The chromosomic location corresponds to the nucleotide in 5’ of the primer.

†Primers used for amplification have been published in Lalani S.R. *et al.*, Am J Hum Genet. 2006 Feb; 78(2):303-314

^#^Primers used for analysis of *CHD7* haplotype.

*CHD7* (NM_017780.4) genomic location (hg. 38) – Chr.8 60678740-60868028.

For amplification of exons 2, 3, 4, 36, and 38, Taq DNA Polymerase (GenScript, Piscataway, NJ, USA) was used. Polymerase chain reaction (PCR) was carried out using one cycle of denaturation at 95^o^ C for two minutes, 35 cycles of denaturation at 95^o^ C for 30 seconds, annealing at 55^o^ C to 60^o^ C, as indicated in the table above for 30 seconds, and extension at 72^o^ C for 1 minute, followed by a final extension at 72^o^ C for 7 minutes.

For amplification of exons 15 and 25 (highlighted in grey), HotStarTaq DNA Polymerase (Qiagen, Hilden, Germany) was used. Touchdown PCR was carried out using one cycle of denaturation at 94^o^C for fifteen minutes, seven touchdown cycles of denaturation at 94^o^ C for 45 seconds, annealing at 58^o^ C for 45 seconds for 7 cycles with 1^o^ C reduction per cycle, and extension at 72^o^ C for 1 minute, 36 cycles of denaturation at 94^o^ C for 45 seconds, annealing at 50^o^ C for 45 seconds, and extension at 72^o^ C for 1 minute, followed by a final extension at 72^o^ C for 2 minutes.

For *CHD7* haplotype, LA Taq Polymerase (TaKaRa Bio Inc, Kusatsu, Shiga, Japan) was used. PCR was carried out using one cycle of denaturation at 94^o^ C for one minute, 30 cycles of denaturation at 98^o^ C for 10 seconds, annealing at 61^o^ C for 30 seconds, and extension at 68^o^ C for 1 minute, followed by a final extension at 72^o^ C for 10 minutes.
